# Measurement of respiratory rate using wearable devices and applications to COVID-19 detection

**DOI:** 10.1038/s41746-021-00493-6

**Published:** 2021-09-15

**Authors:** Aravind Natarajan, Hao-Wei Su, Conor Heneghan, Leanna Blunt, Corey O’Connor, Logan Niehaus

**Affiliations:** Fitbit Research, San Francisco, CA USA

**Keywords:** Endocrinology, Health care

## Abstract

We show that heart rate enabled wearable devices can be used to measure respiratory rate. Respiration modulates the heart rate creating excess power in the heart rate variability at a frequency equal to the respiratory rate, a phenomenon known as respiratory sinus arrhythmia. We isolate this component from the power spectral density of the heart beat interval time series, and show that the respiratory rate thus estimated is in good agreement with a validation dataset acquired from sleep studies (root mean squared error = 0.648 min^−1^, mean absolute error = 0.46 min^−1^, mean absolute percentage error = 3%). We use this respiratory rate algorithm to illuminate two potential applications (a) understanding the distribution of nocturnal respiratory rate as a function of age and sex, and (b) examining changes in longitudinal nocturnal respiratory rate due to a respiratory infection such as COVID-19. 90% of respiratory rate values for healthy adults fall within the range 11.8−19.2 min^−1^ with a mean value of 15.4 min^−1^. Respiratory rate is shown to increase with nocturnal heart rate. It also varies with BMI, reaching a minimum at 25 kg/m^2^, and increasing for lower and higher BMI. The respiratory rate decreases slightly with age and is higher in females compared to males for age <50 years, with no difference between females and males thereafter. The 90% range for the coefficient of variation in a 14 day period for females (males) varies from 2.3–9.2% (2.3−9.5%) for ages 20−24 yr, to 2.5−16.8% (2.7−21.7%) for ages 65−69 yr. We show that respiratory rate is often elevated in subjects diagnosed with COVID-19. In a 7 day window from *D*_−1_ to *D*_+5_ (where *D*_0_ is the date when symptoms first present, for symptomatic individuals, and the test date for asymptomatic cases), we find that 36.4% (23.7%) of symptomatic (asymptomatic) individuals had at least one measurement of respiratory rate 3 min^−1^ higher than the regular rate.

## Introduction

It is well known that heart rate varies with respiration, increasing during inhalation, and decreasing during exhalation. This modulation of the heart rate in response to respiration is known as Respiratory Sinus Arrhythmia (RSA), and is associated with the efficiency of pulmonary gas exchange^1–3^. RSA thus manifests as excess power at the respiration frequency, making it possible to infer the respiratory rate from heart beat interval data.

Unlike other vital signs such as pulse rate and blood pressure, the respiratory rate can be consciously altered by a patient who is aware of the measurement being made, potentially resulting in flawed recordings. The respiratory rate is a valuable metric in determining clinical deterioration^4,5^ and an increase of 3–5 min^−1^ can indicate deterioration^4^. The heart rate to respiratory rate ratio and respiratory rate to oxygen saturation ratio have been shown to be useful indicators in predicting the duration of hospitalization^6^. In a study of patients admitted to the hospital with pneumonia from 2010 to 2012, it was shown that those with a respiratory rate in excess of 27 min^−1^ had an odds ratio of 1.72 for in-hospital death^7^. The respiratory rate factors into the CURB-65 score for predicting mortality in community-acquired pneumonia^8^, as well as during epidemics^9^. Elevated respiratory rate values (>27 min^−1^) have been shown to be predictive of cardiopulmonary arrest^10^. Increased respiratory rate factors into early warning scores meant to assess the likelihood of a patient needing critical care^11–13^. The respiratory rate has also been shown to be a useful biomarker for COVID-19 detection^14,15^. Despite these findings, the respiratory rate is not always recorded while monitoring patients, and may be considered a neglected vital sign^6,16,17^.

The clinical value in measuring respiratory rate, and the growing interest in wearable devices provides a valuable opportunity in the field of digital health. Wearable devices can compute the respiratory rate during sleep, thus obtaining measurements that are made without the conscious knowledge of the user. Commercial wearable devices accomplish this through photoplethysmography (PPG)^18–20^, usually at a single point of contact, either on the wrist (smartwatches, trackers, straps) or the finger (rings). Respiration modifies the PPG time-series signal in a number of ways^21–23^. In this work, we focus on the RSA feature, i.e., the frequency modulation of the PPG.

Karlen et al.^22^ computed the respiratory rate from PPG from short time segments of 32s, with applications in the diagnosis of childhood pneumonia. In a study involving both children and adults, they found agreement with capnometry measurements up to respiratory rates of ~45 min^−1^, and reported a root mean squared error of 3 ± 4.7 min^−1^ over the range of their measurements. Schäfer and Kratky^23^ compared different techniques to estimate respiratory rate from time segments of 5 min, and found a mean absolute error of 0.84 min^−1^ in young subjects, and 1.5 min^−1^ in elderly individuals. Bian et al.^24^ used deep learning techniques to estimate the respiratory rate using PPG, from 1 min long time segments, obtaining a mean absolute error of 2.5 ± 0.6 min^−1^. Shuzan et al.^25^ used a machine learning approach and extracted features from PPG segments of size 32s, to estimate the respiratory rate, with a mean absolute error of 1.91 min^−1^. Dubey et al.^26^ used a spectral kurtosis based method to estimate the respiratory rate from PPG segments of size 32s, yielding a root mean squared error of 1.2 ± 0.3 min^−1^. Dai et al.^27^ have described an algorithm to estimate the respiratory rate on smart watches in the presence of motion. Prinable et al.^28^ employed a Long Short Term Memory architecture to obtain respiratory parameters such as respiratory rate, interbreath interval, inspiration, and expiration time. Berryhill et al.^29^ showed that the respiratory rate computed by WHOOP wearable devices compared well with polysomnography (PSG) measurements during sleep in a study involving 32 participants, with low bias (1.8%) and precision error (6.7%).

In the present work, we describe how the respiratory rate may be inferred from the RSA feature in the power spectral density of heart beat interval time series data. In contrast with several previous works, we restrict our analysis to periods of time when subjects are asleep. We compute power spectra from 5 min segments of data, and average the different 5 min windows over the course of a night. The respiratory rate estimate is made from the averaged power spectral density. We thus obtain a single measurement of respiratory rate, along with an estimated spread, over the course of a night. We compare our measured values with validation data obtained from ground truth measurements, and show that there is good agreement. We examine how the respiratory rate varies with age and sex, and how much it varies relative to the mean value over the course of 14 days. We also investigate its dependence on BMI and heart rate. Finally, we build upon earlier work^15^ and show that longitudinal changes in nocturnal respiratory rate can be a valuable biometric in the detection and monitoring of COVID-19.

## Results

### Sleep stages, heart rate variability, and respiratory rate

Sleep consists of three main stages: Light sleep (stages *N*_1_ and *N*_2_), deep sleep (stage *N*_3_), and REM sleep. Fitbit has developed a validated algorithm that estimates a person’s different sleep stages over a night. Fitbit heart rate and sleep measurements have been studied by an external group who found that Fitbit Charge HR devices showed a 97% sensitivity and a 91% accuracy in detecting sleep^30^. It is known that different stages of sleep are likely to have varying magnitudes of respiratory sinus arrhythmia^31^. We believe that an estimate of respiratory rate during non-REM sleep, or solely taken from deep sleep (*N*_3_) may be more physiologically representative than an all-stages nocturnal respiratory rate. The sinus arrhythmia component is contained within the HF band for respiratory rate values > 9 min^−1^. Thus the HF power can serve as a proxy for the magnitude of sinus arrhythmia. Let us define the dimensionless metric HF_*ν*_ = HF/(HF + LF). The value of HF_*ν*_ averaged over all individuals in deep sleep is found to be HF_*ν*,deep_ = 0.40 ± 0.17 (stated values are mean and standard deviation). In Light sleep, the equivalent HF_*ν*,light_ = 0.27 ± 0.13, while in REM sleep, we find HF_*ν*,REM_ = 0.19 ± 0.11. We thus find with our data that HF power is largest in deep sleep, and least during REM sleep. For the following results, we ignore REM sleep, and estimate the respiratory rate primarily during deep sleep if SNR_deep_ ≥ 2.5 is obtained and during light sleep (provided SNR_light_ ≥ 2.5) if SNR_deep_ < 2.5. We note that in the validation test described in the Methods section, we computed respiratory rate during all sleep stages since we did not have sleep stage information for the data collected with the PSG and home sleep test (HST). A large difference in respiratory rate between sleep stages is not expected according to Ref. ^32–34^. However, Ref. ^35^ found a statistically significant increase from 16.1 ± 2.0 min^−1^ in non-REM sleep to 17.9 ± 2.7 min^−1^ in REM sleep (*p* < 0.05). Ref. ^36^ also found a statistically significant difference in respiratory rate among sleep stages (*p* < 0.001), with REM sleep having the highest rate (*p* < 0.01).

We estimated the probability of the algorithm taking 0, 1, 2, 3, 4, and 5 iterations to estimate the respiratory rate, using a subset of 1000 randomly selected individuals on one night of data (0 iterations means there was either no data, or the signal-to-noise ratio was found to be too low for a reliable estimate. 14.6% of measurements had 0 iterations, i.e., no result with deep sleep data, 6.1% of measurements had no result with light sleep data, and 2.6% of measurements had no result with either deep or light sleep data). For measurements in deep sleep, the fraction of estimates taking 1, 2, 3, 4, 5 iterations were respectively, 50.8%, 22.6%, 4.2%, 1.3%, and 6.5%. For measurements in light sleep, the fractions were found to be 50.7, 28.6, 5.7, 1.2, and 7.7%. These results assume a convergence threshold of 1% between successive iterations. Note that respiratory rate estimates that take five iterations may not have attained the required level of convergence (if the convergence threshold is relaxed to 5%, only 1% of measurements in deep sleep and 0.9% in light sleep required five iterations).

### Variation of respiratory rate with age and sex

**Fig. 1:**
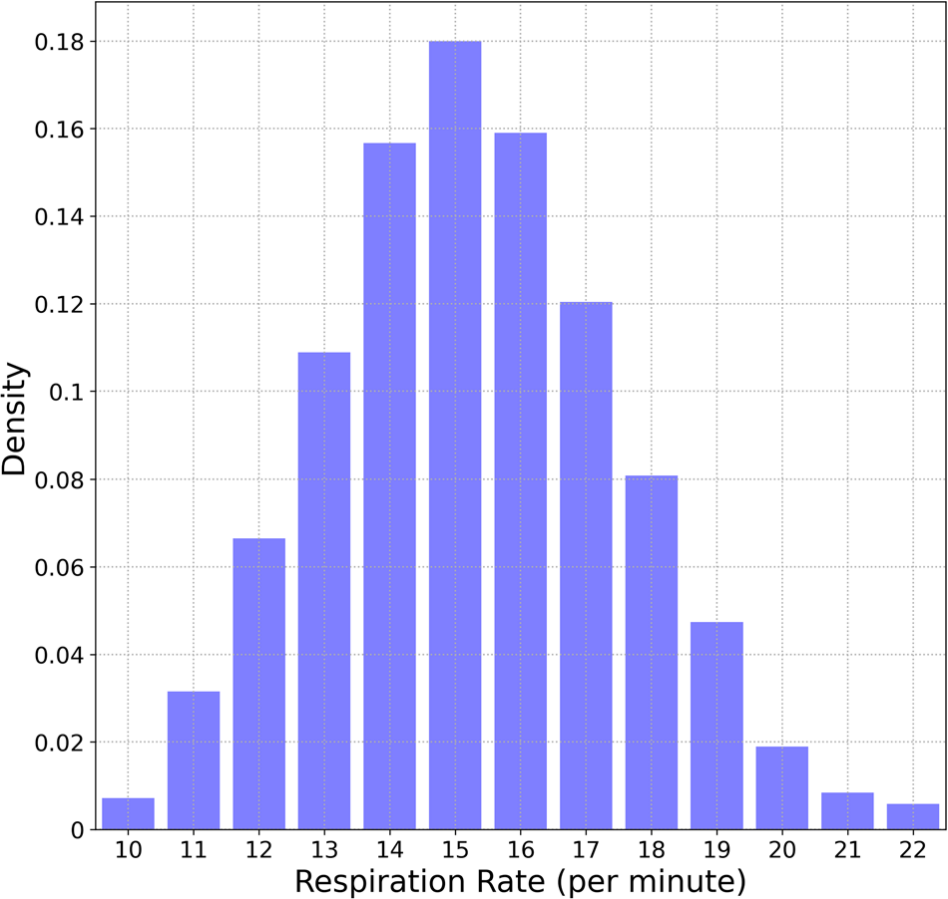
Distribution of respiratory rate. Distribution of average nocturnal respiratory rate. 90% of values are between 11.8 and 19.2 min^−1^.

Figure 1 shows the distribution of respiratory rate values, with a bin size of 1 min^−1^. 90% of values fall in the range 11.8−19.2 min^−1^. The 95% range is 11.2−20.0 min^−1^. The mean of the distribution is 15.4 min^−1^ and the standard deviation is 2.35 min^−1^.

**Fig. 2:**
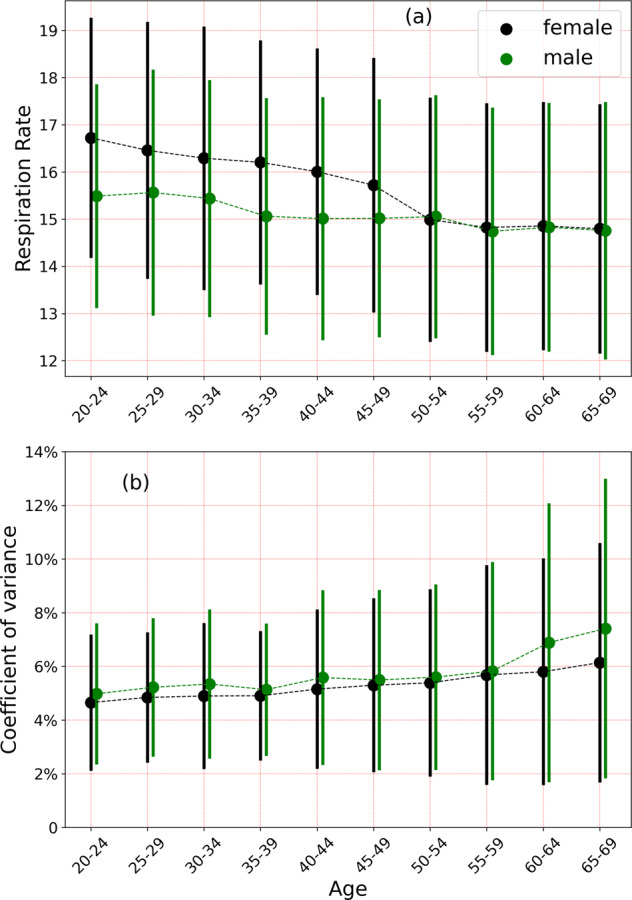
Variation of respiratory rate with age and sex. **a** This shows the variation of respiratory rate with age and sex. Females have a higher respiratory rate on average for ages <50 yr, and no difference thereafter. **b** This shows the coefficient of variation over a 14 day period. Error bars are 1 standard deviation.

Figure 2a shows the variation of respiratory rate with age and sex. The black points show the measurements for female participants, while the green dots represent males. The age bin size is 5 years, and the error bars are one standard deviation. The respiratory rate for females is higher than for males for age <50 yr (*p* value < 0.001). There is no statistically significant difference between males and females for age > 50 yr. The mean respiratory rate for females (males) decreases from 16.7 (15.5) min^−1^ in the age group 20–24 yr, to 14.8 (14.8) min^−1^ in the age group 65–69 yr, a difference of 1.9 (0.7) min^−1^ for females (males) over a span of 50 yr. For age below 50 yr, the Pearson *r* correlation coefficient comparing the dependence of mean respiratory rate with age for females (males) is −0.145(−0.104). For ages >50 yr, we find *r* = −0.031(−0.043) for females (males). Figure 2b shows the coefficient of variance (CoV) (ratio of standard deviation to the mean) measured over a 14 day period, and only considering subjects with 10 or more nights of data. The CoV increases with age, with a Pearson *r*-correlation coefficient of 0.132 (0.172) for females (males). The CoV varies from 4.65% (4.98%) in the age range 20–25 yr to 6.14% (7.41%) in the age range 65–69 yr for females (males). The difference between male and female participants is most significant above age 60 yr (*p* value < 0.001).

### Variation of respiratory rate with BMI and nocturnal heart rate

**Fig. 3:**
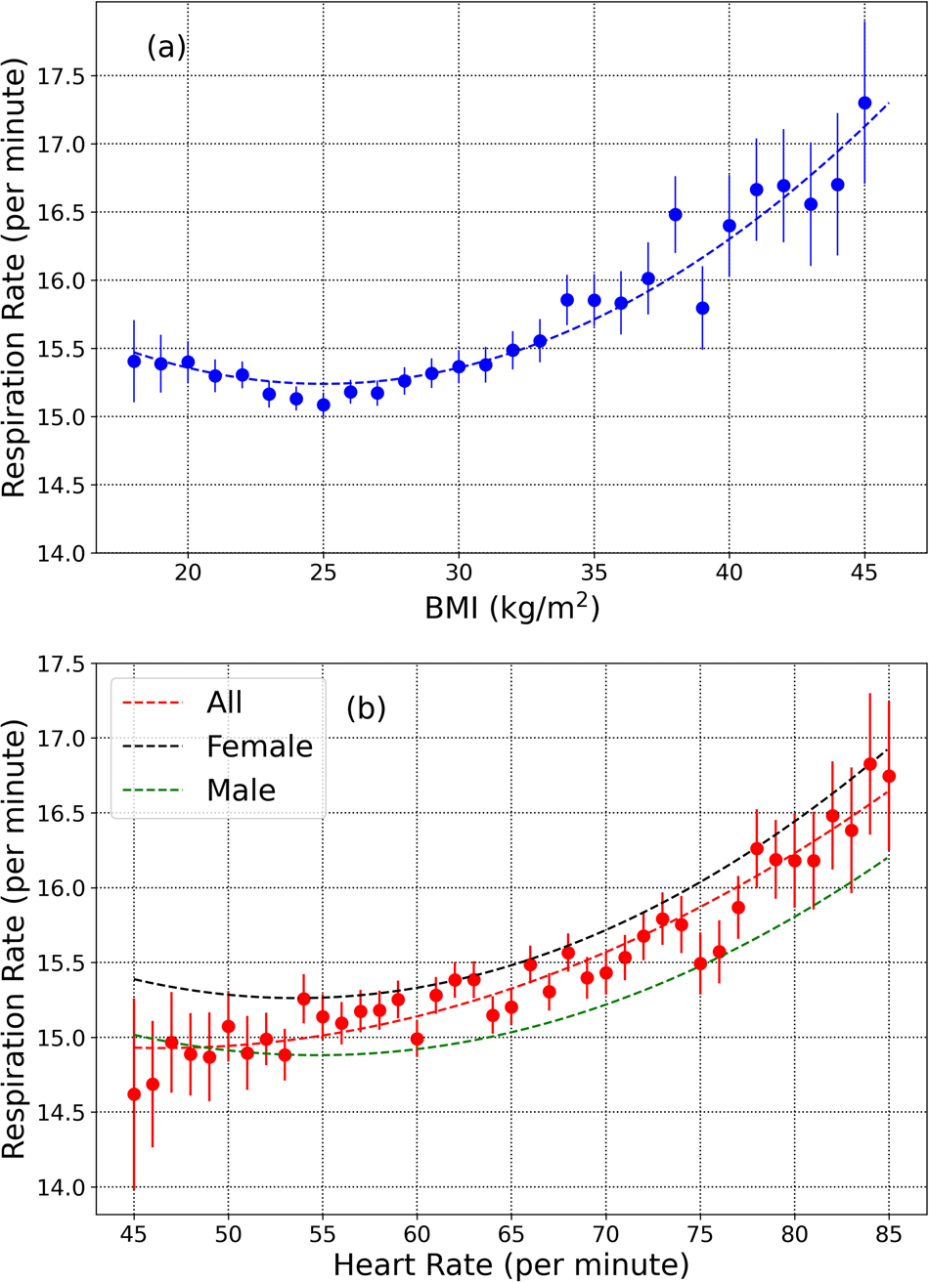
Variation of respiratory rate with BMI and heart rate. **a** Respiratory rate dependence on BMI. The lowest value occurs at a BMI of ~25. **b** Respiratory rate variation with nocturnal heart rate measured in non-REM sleep (black and green curves are for females and males, respectively, the red curve is for all participants). Error bars show the standard error of the mean.

The dependence of respiratory rate with BMI (measured in kg/m^2^) is shown in Fig. 3a. The bin size of BMI = 1 and the error bars represent the standard error of the mean. The respiratory rate reaches a minimum at BMI ≈ 25. For BMI < 25, the respiratory rate decreases with increase in BMI, with a Pearson *r-*correlation coefficient ≈−0.04. For values 25 ≤ BMI < 35, we see an increase with BMI, with *r* ≈ 0.09. For BMI ≥ 35, the correlation coefficient *r* ≈ 0.17. The low correlation is due to a large amount of scatter in the data. Note that the correlation coefficient was determined from all the data points, while Fig. 3 shows the average respiratory rate. Expanding in a Taylor series about the minimum, we find that the mean respiratory rate *R* measured in min^−1^ may be reasonably modeled as:

1 $$R={\alpha }_{{{{\rm{BMI}}}}}+{\gamma }_{{{{\rm{BMI}}}}}\ {\xi }_{{{{\rm{BMI}}}}}^{2},$$

where *α*_BMI_ = 15.24, *γ*_BMI_ = 2.95. ${\xi }_{{{{\rm{BMI}}}}}=\frac{{{{\rm{BMI}}}}-25}{25}$. Eq. (1) is a useful model over the range of BMI 18−45. Eq. (1) was fitted to the data points shown in Fig. 3a in the range 18−45 and the fit is shown by the dotted line (coefficient of determination *R*^2^ = 0.902).

The variation of respiratory rate with nocturnal heart rate is shown in Fig. 3b. The heart rate in beats per minute (bpm) is measured in non-REM sleep. The mean respiratory rate (for all participants) increases with increase in heart rate. For average nocturnal heart rate values *HR* < 65 bpm, the Pearson *r-*correlation ≈ 0.07, while for *H* ≥ 65 bpm, we find *r* ≈ 0.14. It is possible to model the mean respiratory rate *R* (measured in min^−1^) dependence on heart rate as:

2 $$R={\alpha }_{{{{\rm{HR}}}}}+{\beta }_{{{{\rm{HR}}}}}{\xi }_{{{{\rm{HR}}}}}+{\gamma }_{{{{\rm{HR}}}}}\ {\xi }_{{{{\rm{HR}}}}}^{2},$$

where *α*_HR_ = 15.14, *β*_HR_ = 1.88, *γ*_HR_ = 4.17. ${\xi }_{{{{\rm{HR}}}}}=\frac{{{{\rm{HR}}}}-60}{60}$. Eq. (2) was fitted to the data points shown in Fig. 3b for all participants (male and female), and is useful over the range 45−85 bpm (coefficient of determination *R*^2^ = 0.915). The black and green dashed lines shown in Fig. 3b are plotted for female and male participants respectively.

### Effect of COVID-19 on nocturnal NREM respiratory rate

**Fig. 4:**
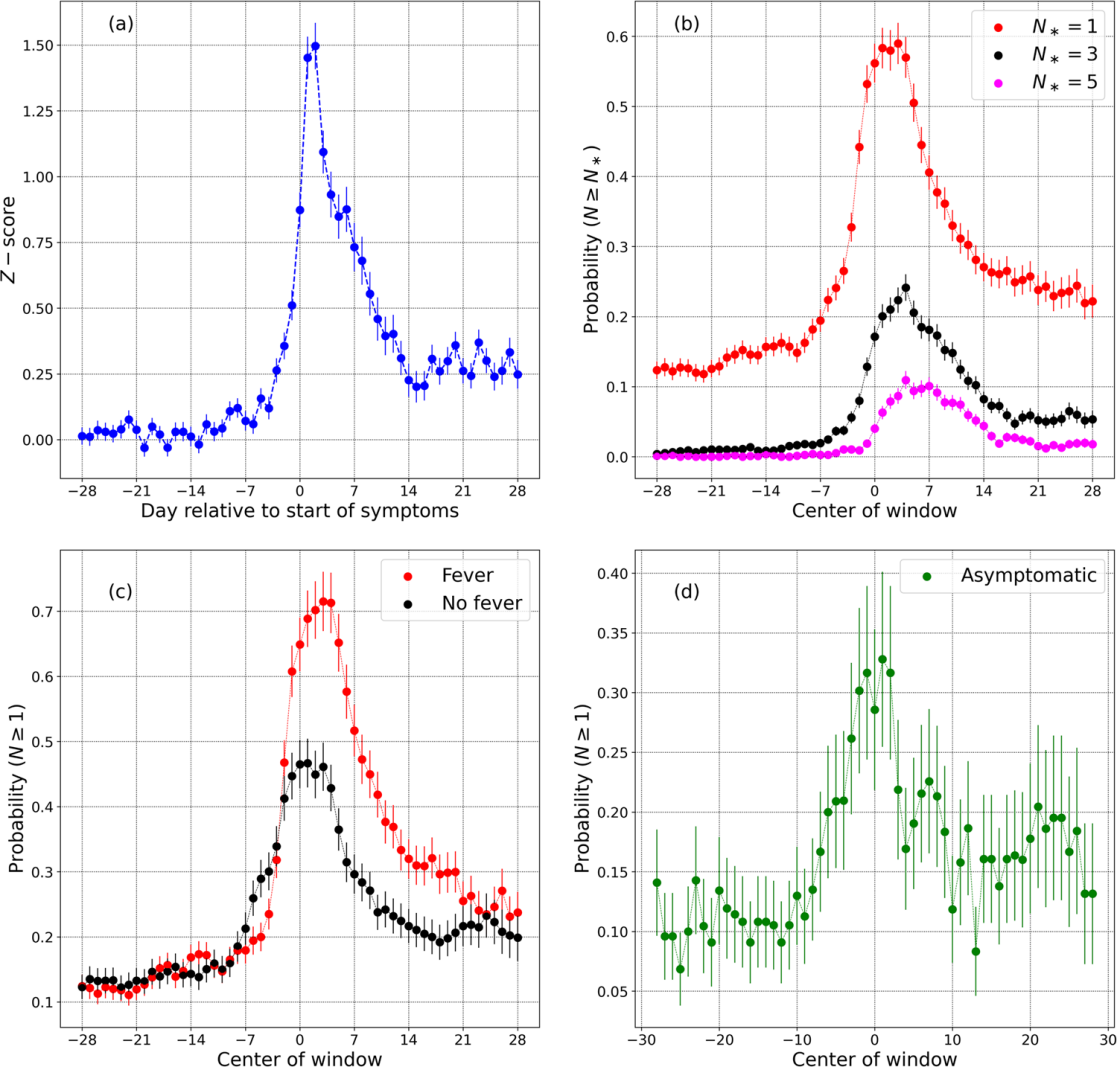
Impact of COVID-19 on respiratory rate. **a** This shows the dimensionless *z-*scored respiratory rate in symptomatic individuals, with day relative to the start of symptoms (Day 0 is the day when symptoms present). **b** Measures the probability of receiving *N* ≥ N_*_ anomalously high values in a 7 day window centered on day *D*, for N_*_ = 1, 3, 5. The effect of fever is seen in **c**. The variation of respiratory rate for asymptomatic individuals is shown in **d**. Error bars show the standard error of the mean.

In this section, we present results from a subset of the Fitbit COVID-19 data survey. Let *μ* and *σ* be the mean and standard deviation of the respiratory rate for a specific user, estimated several days prior to the onset of illness. The *Z* − score on a given day *D*_*n*_ may be defined as

3 $$Z({D}_{n})=\frac{R({D}_{n})-\mu }{\sigma },$$

where *R*(*D*_*n*_) is the respiratory rate for a specific user on day *D*_*n*_. For symptomatic individuals, let *D*_0_ be the date when symptoms present. For asymptomatic users, we set *D*_0_ to the test date. Mean and standard deviation of the respiratory rate are computed using data from *D*_−90_−*D*_−30_, only considering users with at least 30 days of data in this date range. There were 1247 symptomatic individuals (from a total of 2939) and 133 asymptomatic individuals (from a total of 297) satisfying this requirement. Figure 4a shows the average *z-*score measured for symptomatic individuals. The *Z*-score ≈ 0 for days <*D*_−14_, but increases thereafter, reaching a peak on *D*_+2_, i.e., two days following the day when symptoms first present. Interestingly *Z* does not fall off to zero, but instead approaches a constant between *D*_+__14_ and *D*_+28_.

Next, we investigate the likelihood that a randomly selected symptomatic individual will receive an anomalously high respiratory rate value on a specific day. Let us consider a 7 day window, and compute the probability that a subject will receive *N* ≥ N_*_ respiratory rate values satisfying *Z* ≥ 2.326 (this threshold corresponds to a *p*-value of 0.01 for a 1-tailed test. We are only concerned with values above the mean). Figure 4b shows the results for 7-day windows centered from *D*_−28_ to *D*_+28_, only considering subjects with all 7 days of valid data in the window. Shown are probabilities for N_*_ = 1, 3, and 5. Figure 4c shows the effect of fever which is known to increase the respiratory rate^37^. The red data points show the probability for N_*_ = 1 for symptomatic individuals who presented with a fever, while the black data points show the same probability for individuals who did not list fever as a symptom. Figure 4d considers the respiratory rate measured for asymptomatic individuals. The plot shows the probability for N_*_ = 1, as a function of window center. In all cases, the error bars represent the standard error of the mean. For plots (b), (c), and (d), we approximated the standard error of a count as the square root of the count.

The increase in respiratory rate may also be quantified by means of the effect size. Let us consider data from symptomatic individuals, in a 7 day window several days prior to the appearance of symptoms. The period from *D*_−24_ to *D*_−18_ serves as the control. Let us also consider a 7 day window during which symptoms are likely to manifest, i.e., *D*_−1_ to *D*_*+*__5_. The effect size (Cohen’s *d*) for each individual may be computed as^38,39^﻿:

4 $$d =\frac{{\mu }_{1}-{\mu }_{2}}{\sigma },$$

where *μ*_1_ and *μ*_2_ are the mean respiratory rate values for the two time windows, and *σ* is the pooled standard deviation:

5 $$\sigma =\sqrt{\frac{\left({N}_{1}-1\right){\sigma }_{1}^{2}+\left({N}_{2}-1\right){\sigma }_{2}^{2}}{{N}_{1}+{N}_{2}-2}}.$$

**Fig. 5:**
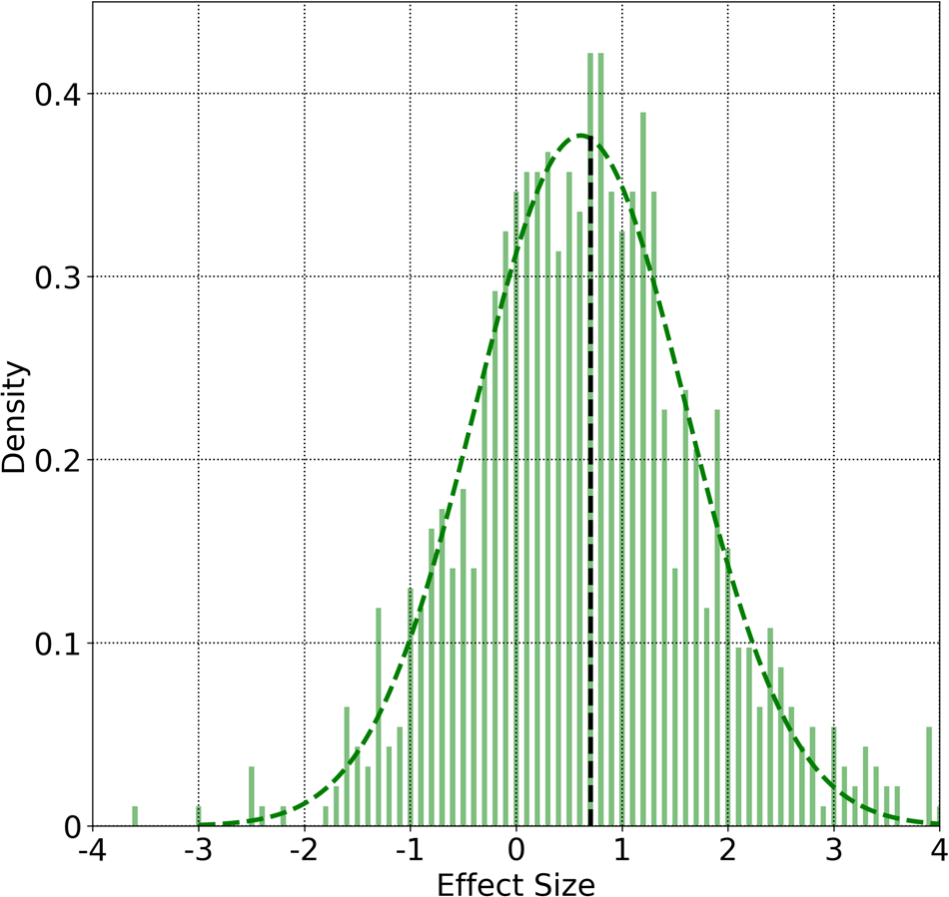
Effect size distribution. Distribution of Cohen’s *d* effect sizes comparing respiratory rates in two-time windows, for symptomatic individuals: A 7-day period from *D*_−24_ to *D*_−18_, and a 7 day window from *D*_−1_ to *D*_*+*__5_. We only consider subjects with at least 5 days of data in the 7 day period. The curves are normalized to unit area. Also shown is a gaussian fit to the data. The vertical line indicates the mean value of the effect size distribution (+0.70).

*N*_1_ and *N*_2_ are the number of days of data in each window. ${\sigma }_{1}^{2}$ and ${\sigma }_{2}^{2}$ are the variances of the data in the two windows. We computed the effect size for each individual (provided they had at least 5 days of data in the selected 7 day period) and the distribution of effect sizes is shown in Fig. 5 (normalized to unit area). The mean effect size is +0.70 and the standard deviation is 1.2.

**Fig. 6:**
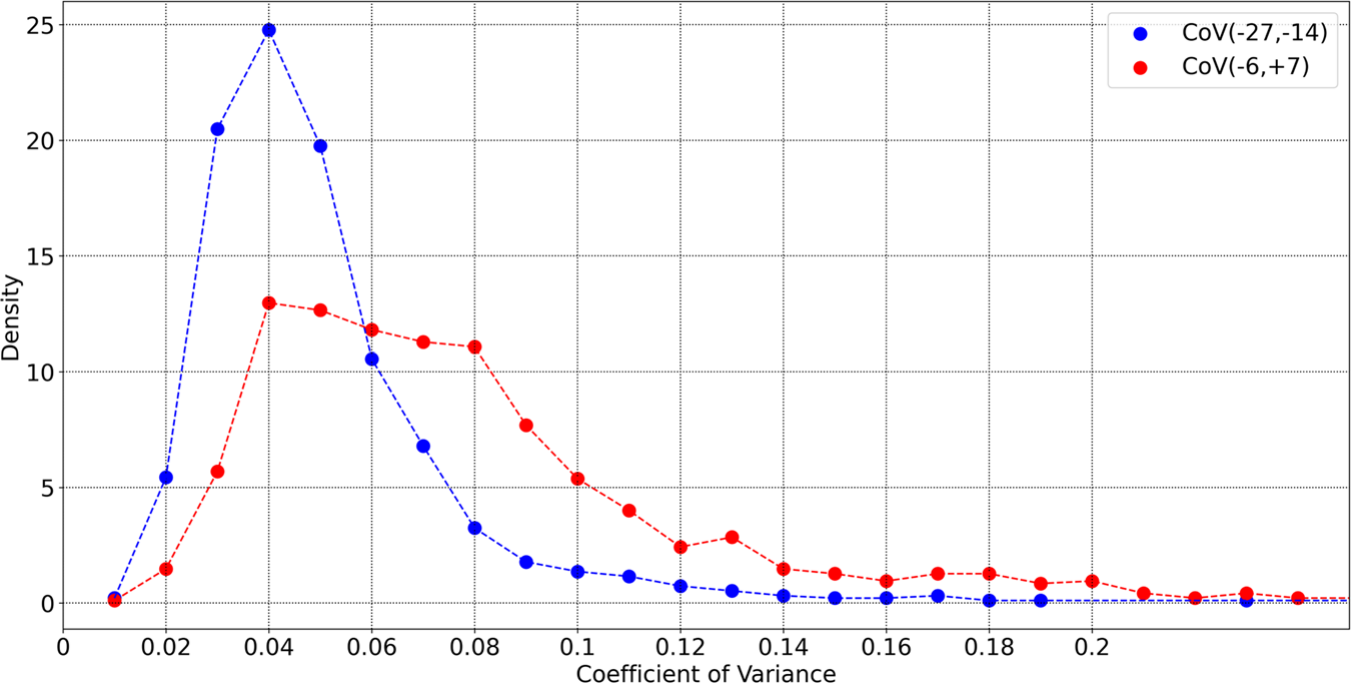
Distribution of coefficient of variance. Distribution of coefficient of variance, for symptomatic individuals. The blue points are for the 14 day time window (*D*_−27_−*D*_−14_), while the red points are computed for the time period (*D*_−6_−*D*_+7_). We only consider subjects who have at least 10 days of the data in the 14 day window. The curves are normalized to unit area.

We have earlier computed typical values of coefficient of variance (CoV) over a 14 day period and found mean values in the range 4–6% for age <60 yr. It is instructive to compare the CoV of healthy individuals with those with symptomatic COVID-19. We use data in the date range *D*_−27_−*D*_−14_ to simulate a 14 day healthy period. Let us also consider the date range *D*_−6_−*D*_+7_ as a 14 day period during which COVID-19 symptoms may affect biometrics. We computed the CoV for each individual in these time windows provided they had 10 or more data points in the selected window. The comparison of the CoV for these periods is shown in Fig. 6 (both curves are normalized to unit area). The blue curve shows the CoV when the participants are presumed healthy, and peaks around ≈4% (mean = 5.05%, std dev = 2.7%). The red curve is the CoV during illness, and shows a larger spread in values compared to the blue curve (mean = 7.9%, std dev = 4.3%).

## Discussion

The key findings of the present work may be summarized as follows:

1. The respiratory rate during sleep may be reliably obtained from photoplethysmography using a smartwatch or tracker. 90% of nocturnal respiratory rate values lie in the range 11.8−19.2 min^−1^.
2. There is a statistically significant difference in respiratory rates between male and female subjects for age <50 yr, with no difference in sex thereafter.
3. The coefficient of variance in a 14 day period is small, with mean values between 4% and 6% for age <60 yr. The coefficient of variance shows a statistically significant difference between males and females for age > 60 yr with no difference in sex for younger individuals.
4. There exists a correlation between respiratory rate and nocturnal heart rate, and with body mass index (BMI). On average, the respiratory rate is elevated for larger values of nocturnal heart rate, and for larger values of BMI. The respiratory rate is also slightly elevated for underweight individuals (low BMI).
5. On average, the respiratory rate is elevated during illness. Using COVID-19 as an example, we found that 36.4% (23.7%) of symptomatic (asymptomatic) individuals had a respiratory rate ≥3 min^−1^ in excess of their mean value in the time window *D*_−1_–*D*_+5_ (where *D*_0_ is the date when symptoms present for symptomatic individuals, or the test date for asymptomatic cases). Comparing respiratory rates for symptomatic individuals in the time window *D*_−__1_−*D*_+5_ to values in the period *D*_−24_−*D*_−18_, we find a mean effect size of +0.70, implying that the median value of respiratory rate during illness would rank at the 76th percentile among respiratory rate values in the healthy period^40^.

We computed the power spectral density from the heart rate interbeat interval time series every 5 min. These individual spectra were then aggregated over a night, and the respiratory rate was estimated from the averaged power spectral density. We validated our technique with the help of nasal cannula data consisting of 52 measurements obtained from 28 participants with apnea-hypopnea index < 30. The bias (mean of the predicted rate—true rate) was found to be −0.244 min^−1^ (−1.67%) while the RMS error was 0.648 min^−1^ (4.18%). The mean absolute error was 0.460 min^−1^, and the mean absolute percentage error was 3%. The absolute value of bias is larger for low values of respiratory rate. For rates lower than 16 min^−1^, the bias is −0.41 min^−1^, while for rates ≥16 min^−1^, the bias is 0.

We also reported on respiratory rate data for 10,000 participants, ranging in age from 20 to 69 years, for both male and female participants. Respiratory rates measured in deep sleep (or light sleep when deep sleep data was unavailable) for adults commonly ranges from 11.8 min^−1^ - 19.2 min^−1^ (90% range, see Fig. 1). For both males and females, respiratory rate values are inversely correlated with age. From ages 20 to 50 yr, the Pearson *r* correlation coefficient for female (male) participants was found to be −0.145(−0.104), while for ages > 50 yr, the corresponding values for females (males) was −0.031(−0.043). The coefficient of variation on the other hand, increases with age (Fig. 2b). The coefficient of variation is higher in males compared to females, for ages greater than 60, with no difference for age <60 yr. From age 20–24 yr, the coefficient of variation measured over a 14 day period range for female (male) participants ranges from 2.3 to 9.2% (2.3–9.5%) (90% range). For subjects in the age range 65−69 yr, the 90% ranges for female (male) participants are 2.5–16.8%(2.7–21.7%). Respiratory rate varies with BMI, reaching a minimum at a BMI of 25 kg/m^2^. It also varies with heart rate, increasing with increase in heart rate measured during non-REM sleep. We note however that BMI and heart rate are not independent of each other^41^.

We see an interesting behavior in the way the respiratory rate varies with age for female and male participants (see Fig. 2a). Female subjects have a higher respiratory rate than males for age < 50 yr, while for age > 50 yr, there is no difference between males and females. Female participants on average, have a higher heart rate than males^42^, and we have shown that the respiratory rate is elevated in individuals with a higher heart rate (Fig. 3b). To determine whether the increased heart rate in females could contribute to the increased respiratory rate, we use Eq. (2) to obtain

6 $${{\Delta }}R={{\Delta }}{H}_{60}\left[{\beta }_{{{{\rm{HR}}}}}+2{\gamma }_{{{{\rm{HR}}}}}\left({H}_{60}-1\right)\right],$$

where HR is the heart rate, *H*_60_ = HR/(60 bpm), *β*_HR_ and *γ*_HR_ are as defined in Eq. (2), and *R* is the mean respiratory rate for individuals with a heart rate HR. For the age group 20−24 yr, we find those male participants have 〈*H*_60_〉 = 1.0031. For female participants in the same age group, we find 〈*H*_60_〉 = 1.1123, giving us 〈Δ*H*_60_〉 = 0.1092. The correlation between heart rate and respiratory rate implies that the increased heart rate can account for at most an excess of Δ*R* ≈ 0.208 min^−1^. The true difference in respiratory rates between females and males in this age group is 1.2 min^−1^ (Fig. 2(a)). The increased heart rate in females can thus account for only 17.3% of the difference between the respiratory rates of females and males. As a further test, we considered heart rate bins of 5 bpm, and selected male and female individuals within the same age bin, and the same heart rate bin. With 280 female, and 357 male participants in the heart rate bin 57.5−62.5 bpm, and the age bin 20−24 yr, we find a mean respiratory rate of 16.5 min^−1^ for females, and 15.6 min^−1^ for males, with an effect size of 0.38, and a *p*-value of 1.54 × 10^−6^. Similar computations can be made for other heart rate bins and age groups. While the effect size is slightly decreased compared to the case where the heart rate is unrestricted, the increased nocturnal heart rate in females cannot solely explain the increase in respiratory rate. A striking feature seen in Fig. 2(a) is the rapid decrease in the mean respiratory rate in female participants around the age ≈50 yr. This leads us to hypothesize that sex hormones are responsible for the difference in respiratory rates between men and women. It is well known that some sex hormones such as progesterone act as respiratory stimulants^43–45^. Since progesterone secretion decreases after menopause^43,45^, it is likely that the change in mean respiratory rate seen in females at age ≈50 yr is associated with menopause.

Finally, we studied how respiratory rate is affected by COVID-19. We computed respiratory rates for 3236 uses of Fitbit devices with test dates ranging from Feb 28–Nov 13, 2020, consisting of 2939 symptomatic and 297 asymptomatic individuals. Let *D*_0_ be the data when symptoms first present, for symptomatic individuals, and the date when the COVID-19 test was taken, for asymptomatic individuals. We estimated the mean and standard deviation of the respiratory rate from *D*_−90_ to *D*_−30_, only considering individuals with 30 or more days of data within this date range. We obtained the mean and standard deviation for 1247 symptomatic individuals (677 who presented with a fever, and 570 who did not) and 133 asymptomatic individuals. The *Z* − scores for each day from *D*_−28_ to *D*_+28_ are shown in Fig. 4(a) averaged over participants. For days up to *D*_−14_, the *Z*-scores are consistent with zero, but increase thereafter, reaching a maximum around ~*D*_+2_. The *Z-*scores decrease for larger *D*_*n*_, but interestingly, they do not fall to zero.

In Fig. 4b, we computed the probability of obtaining *N* ≥ N_*_ measurements satisfying *Z* ≥ 2.326. Let us estimate the noise floor by averaging the probability in the 14 day period *D*_−28_ ≤ *d* < *D*_−14_. For N_*_ = 1,3, and 5, we find noise floor values equal to 13.4%, 0.88%, and 0.092%, while the peak values are respectively, 59.3%, 23.9%, and 11.1%, yielding peak-to-noise ratios of 4.42, 27.1, and 120.4 respectively. Setting the noise floor as the false positive rate, and assuming a disease prevalence of 1 per 1000 individuals per day, we obtain positive predictive values for N_*_ = 1,3,5 to be 0.440%, 2.641%, and 10.76% respectively. For symptomatic individuals presenting with a fever (Fig. 4(c)), the *P*(*N* ≥ 1) plot peaks at 71.5%, while for symptomatic individuals who do not present with a fever, the plot peaks at 47.3%. For asymptomatic individuals (Fig. 4(d)), the plot for N_*_ = 1 peaks at 33.3%. This is smaller than for symptomatic individuals (59.3%) and for individuals who present with a fever (71.5%).

Considering the excess respiratory rate Δ_*R*_ = respiratory rate for a user on a given day relative to the normal value for that user, we find that 36.4% (23.7%) of symptomatic (asymptomatic) individuals recorded a value of Δ_*R*_ ≥ 3 min^−1^ on at least one day in the 7 day window from *D*_−1_ and *D*_+5_ (as before *D*_0_ is the date when symptoms present, for symptomatic individuals, and the test date for asymptomatic individuals). For a larger excess respiratory rate Δ_*R*_ ≥ 5 min^−1^, the equivalent percentages for symptomatic (asymptomatic) individuals are 12.3% (5.1%). It is instructive to compare these numbers to the excess respiratory rate expected in a 7 day window, for a population of healthy individuals. Since we do not have a separate dataset of confirmed healthy individuals, let us examine the subjects in the symptomatic COVID-19 dataset on dates far before the start of symptoms. When measured during the 7 day window from *D*_−24_ to *D*_−18_, only 4.3% of individuals received one or more values of Δ_*R*_ ≥ 3 min^−1^, and only 1.05% showed one or more values with Δ_*R*_ ≥ 5 min^−1^. Comparing the respiratory rate in the two time periods, we find a range of effect sizes with a mean of + 0.70 and a standard deviation of 1.2. This implies that an average respiratory rate value in the sick window would be greater than 76% of respiratory rate values in the control window^40^. The distribution of effect sizes is show in Fig. 5. We also showed that the coefficient of variance shows a broader distribution when computed during illness (Fig. 6).

There are several limitations to the present work. The technique we have described cannot be applied when there are motion artifacts, i.e., when subjects are moving about. The algorithm only generates one estimate of respiratory rate over the whole night (as well as a measure of the spread), and we cannot, therefore, make accurate estimates of quantities such as respiratory rate variability. The signal quality derived from PPG is also likely to be inferior to ECG from a chest strap, although trackers and smartwatches are far more comfortable to wear. The dataset of 10,000 participants consisted of individuals who were randomly selected. We did not attempt to exclude subjects with significant sleep apnea (for whom "average” respiratory rate may be hard to define). Age, sex, and BMI were provided by the user, and we are unable to verify these demographic data. We have assumed that participants were healthy during the 2 week period of study, but we do not have evidence of this. This limitation is even more important for the COVID-19 study. Although we have assumed that individuals are healthy several days prior to being diagnosed with COVID-19, we do not have any way to confirm this. The date of COVID-19 diagnosis was provided by the participants themselves, and errors in this date can affect our results. Nevertheless, the results presented in this work establish that respiratory rate is a valuable health metric that can be reliably computed using wearable devices.

## Methods

This study uses three data sets (see Table 1 for a summary) to both validate the core algorithm, and to report on interesting population and subject level longitudinal trends. We first explain the origin and scale of the three data sets.

**Table 1:** Details of datasets considered in this study.

Name of Dataset	Number of individuals	Demographics	Dates	Purpose
Sleep study respiratory rate validation data set	28	Mean age: 48.9 yr (53.6% fem.)	Oct 17−Nov 6, 2019 & Mar 9−May 29, 2020	To obtain respiratory rate ground truth data for validation of the algorithm.
General population nocturnal respiratory rate data set	10,000	Ages 20−69 yr (50% fem.)	Nov 1−14, 2020	To study respiratory rate variation with age and sex, to establish transverse benchmarks, and to study longitudinal variability.
Longitudinal nocturnal respiratory rate data set from COVID-19 subjects	3,236	Mean age: 42.25 yr (77.6% fem.)	Feb 28−Nov 13, 2020	To collect COVID-19 information from Fitbit users, including symptoms and dates.

### Sleep study respiratory rate validation data set

We conducted 2 experiments to validate the respiratory rate algorithm. Experiment A was conducted at Sleep Med in Columbia, SC, from Oct 17, 2019 to Nov 6, 2019, and used a polysomnography device (Alice 5). Experiment B was conducted remotely, by shipping equipment to the homes of participants, from March 9, 2020 to May 29, 2020, and used a Home Sleep Test (Resmed Apnealink). Both experiments were approved by an Institutional Review Board (Solutions IRB). Participants provided informed consent for their data to be collected and used for research purposes. Participants in Experiment A wore Fitbit devices on both wrists, while participants in Experiment B wore a Fitbit device on one wrist only. We excluded participants with severe sleep apnea (Apnea-Hypopnea Index ≥ 30). 52 measurements were obtained from 28 individuals (15 female, 13 male) between the ages of 32 and 71 (mean age was 48.9 yr with a standard deviation of 9.5 yr). More details regarding the data collection may be found in Supplementary Table I.

### General population nocturnal respiratory rate data set

The dataset used to explore correlations between respiratory rate and age, sex, BMI, and heart rate consisted of 10,000 users of Fitbit devices who reside in the United States or Canada, and who wore their devices to sleep in the date range Nov 1–14, 2020. We collected sleep and heart rate variability data from these Fitbit users during this 14 day period. The data were collected and anonymized consistent with Fitbit’s terms and conditions. The dataset consisted of male and female individuals in the age range 20–69 with 500 subjects of each sex and each of 10 equally spaced age bins (5 year age bin size), yielding a total of 135,947 usable measurements. The mean Body Mass Index (BMI) of the participants was 27.8 ± 5.2 for males and 27.5 ± 6.4 for females, where the quoted error bar is 1 standard deviation. The main Fitbit devices used to collect these data include Charge 3 (22.5%), Versa 2 (20.0%), Inspire HR (11.3%), Versa (10.0%), Charge 2 (9.62%), and Charge 4 (7.68%), with a number of other devices contributing <5% each.

### Longitudinal nocturnal respiratory rate data set from COVID-19 subjects

The Fitbit COVID-19 survey is an ongoing survey of Fitbit users residing in the United States or Canada. Participants provide information on whether they were diagnosed with COVID-19, and whether they experienced symptoms. The data for the COVID-19 survey were collected with Institutional Review Board approval (Advarra IRB), and participants provided written consent for their data to used for research purposes. The data used in the present study comprises a subset, consisting of 3236 individuals with COVID-19 PCR positive test dates (self-reported) ranging from Feb 28–Nov 13, 2020, with 2,939 symptomatic and 297 asymptomatic individuals. 77.6% of participants identified as female. The mean age was 42.25 ± 12.35 yr, and the mean BMI was 30.29 ± 7.25, where the stated errors are 1 standard deviation. More details regarding the Fitbit COVID-19 survey may be found in Ref. ^15^.

### Software

All statistical analyses were performed using standard Python packages such as NUMPY and SCIPY. The respiratory rate code software was written in Scala and uses the BREEZE library.

### Computation of heart rate variability

**Fig. 7:**
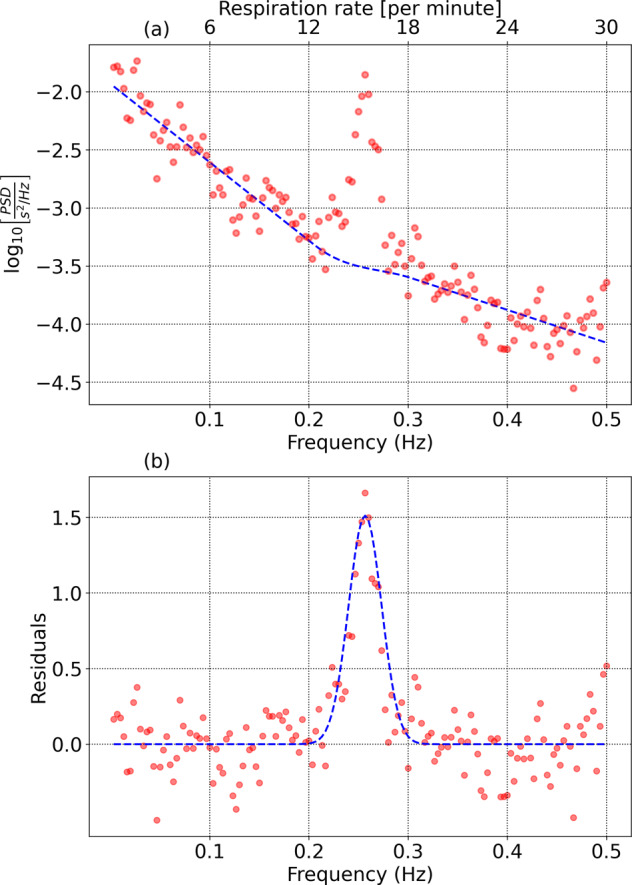
Isolating the RSA component from the PSD. **a** HRV power spectral density consisting of background and Respiratory Sinus Arrhythmia. **b** Residuals after the background are subtracted.

Interbeat interval values are computed from the heart beat interval time series data and assembled into non-overlapping 5 min blocks. The data are cleaned to remove noise due to motion artifacts, electronic artifacts, missed heart beats, etc. For details on the cleaning and pre-processing steps, we refer the reader to Ref. ^46^. Each 5 min block of data is resampled to obtain 512 equally spaced samples allowing us to resolve all frequency components up to 0.5 × (512/300) = 0.85 Hz. The resolution in frequency space is 1/300 Hz. The mean of the data in the time window is subtracted, and the data smoothed with a Hann window. A Fast Fourier Transform is applied, and properly normalized to give us the Power Spectral Density (PSD), which is the power contained per unit frequency. Integrating the PSD over the range 0.04 Hz - 0.15 Hz gives us the low frequency (LF) power, while integrating the PSD over the range 0.15–0.4 Hz gives us the high frequency (HF) power. The PSD for different 5 min segments are aggregated. The PSD of HRV fluctuations is shown in Fig. 7 for a single individual and for one night: The plot contains two main components: background and RSA. To isolate the RSA component, we need to model the background and subtract it from the power spectrum.

### Modeling the background

We set a maximum frequency ${f}_{\max }$ = 0.5 Hz (corresponding to a respiratory rate of 30 min^−1^), and discard data at higher frequencies. We also set a minimum frequency ${f}_{\min }$ = 0.1367 Hz (corresponding to a respiratory rate of 8.2 min^−1^). The power spectrum at frequencies from *f*_0_ = 1/300 Hz to ${f}_{\min }$ is used to determine the noise level. The RSA feature is contained between two frequencies ${f}_{1}( > {f}_{\min })$ and ${f}_{2}( < {f}_{\max })$ which we will determine iteratively.

1. Low frequency background: The PSD from frequencies *f*_0_ to *f*_1_ is modeled by a function of the form ${{{\mathrm{log}}}\,}_{10}[{{{\rm{PSD}}}}]={c}_{1}+{c}_{2}f$.
2. High frequency background: The PSD from frequencies *f*_2_ to ${f}_{\max }$ is modeled by a similar function: ${{{\mathrm{log}}}\,}_{10}[{{{\rm{PSD}}}}]={c}_{3}+{c}_{4}f$.
3. The PSD from *f*_1_ to *f*_2_ is modeled by a *patching function*: ${{{\mathrm{log}}}\,}_{10}[{{{\rm{PSD}}}}]={p}_{1}+{p}_{2}f+{p}_{3}{f}^{2}+{p}_{4}{f}^{3}$. The parameters *p*_1_, *p*_2_, *p*_3_ and *p*_4_ are fixed to match the end points of the low frequency and high frequency background regions, as well as the derivatives at the end points, thus enabling a smooth transition.

### Isolating the signal

To begin, we assign reasonable values to *f*_1_ and *f*_2_, which will be refined in subsequent iterations. We initialize ${f}_{1}={f}_{\min }$ and *f*_2_ = 0.333 Hz (corresponding to a respiratory rate of 20 min^−1^). In practice, the choice of *f*_1_ and *f*_2_ are determined by the expected range of respiratory rates in the population under study. Signal estimation is performed using the following steps:

1. The power spectrum is modeled as described earlier, and parameterized by the variables (*c*_1_, *c*_2_, *c*_3_, *c*_4_, *p*_1_, *p*_2_, *p*_3_, *p*_4_).
2. The background function is subtracted from the data to obtain the residuals. The residuals are low pass filtered (we use a median filter of size 3) to reduce noise, and interpolated (we use a cubic spline) to maintain the original frequency resolution.
3. The peak of the residuals is identified as *A*_RSA_, and the frequency corresponding to the maximum value = *f*_RSA_. Assuming a gaussian distribution for the RSA feature, we identify a frequency *f*_−_ < *f*_RSA_ such that *A*(*f*_−_) = 0.6065*A*_RSA_, as well as a frequency *f*_+_ > *f*_RSA_ such that *A*(*f*_+_) = 0.6065*A*_RSA_. The mean of these two values *f*_resp_ = 0.5 × (*f*_+_ + *f*_−_) is identified as the mean respiratory frequency. The standard deviation is *σ*_resp_ = 0.5 × (*f*_+_ − *f*_−_). The mean *μ*_noise_ and standard deviation *σ*_noise_ of the residuals from *f*_0_ to ${f}_{\min }$ are calculated. The signal-to-noise ratio *SNR* is defined as $SNR=\left({A}_{{{{\rm{RSA}}}}}-{\mu }_{{{{\rm{noise}}}}}\right)/{\sigma }_{{{{\rm{noise}}}}}$.
4. *f*_1_ is redefined as *f*_resp_ − 3*σ*_resp_, and *f*_2_ is set to *f*_resp_ + 3*σ*_resp_.

Steps 1–4 are repeated until either successive estimates of *f*_resp_ agree to within 1%, or 5 iterations are completed. We restrict our range of respiratory rates to between 10 and 26 min^−1^. Frequencies much higher than 26 min^−1^ are hard to resolve due to the rapid fall-off of the power spectral density with frequency, while resonances at frequencies lower than 10 min^−1^ may be confused with Mayer wave oscillations^47^. The values of (*f*_resp_, *σ*_resp_, *SNR*) are stored for each individual, for each day, provided SNR ≥ 2.5. Figure 7b shows the residuals and estimation of the RSA feature. Also shown is a gaussian with mean *f*_resp_ and standard deviation *σ*_resp_.

When aggregating respiratory rate measurements over multiple days, we adopt a numerical approach: The respiratory rate measurement for any given day for each individual is treated as a random variable drawn from a gaussian distribution with mean *f*_resp_ and standard deviation *σ*_resp_. We randomly choose 100 samples from this distribution for each day. The mean and standard deviation over all samples is then computed. We follow the same process for averages involving multiple subjects.

### Validation of estimated respiratory rate data with ground truth measurements

We obtained 52 measurements of airflow data, from 28 individuals through PSG, or a HST. Data were collected from 1 to 3 nights for each participant, with devices on either one or both wrists (data from the two experiments were combined, see Supplementary Table I for details). Data from the air flow sensor were band pass filtered with a fourth-order Butterworth filter to retain frequencies between 10 and 30 min^−1^. The data were then analyzed with the help of a spectral peak detection algorithm with a window size of 51.2s and a step size of 6.4s. The median of all respiratory rate measurements over the night is computed, and serves as the true respiratory rate.

**Fig. 8:**
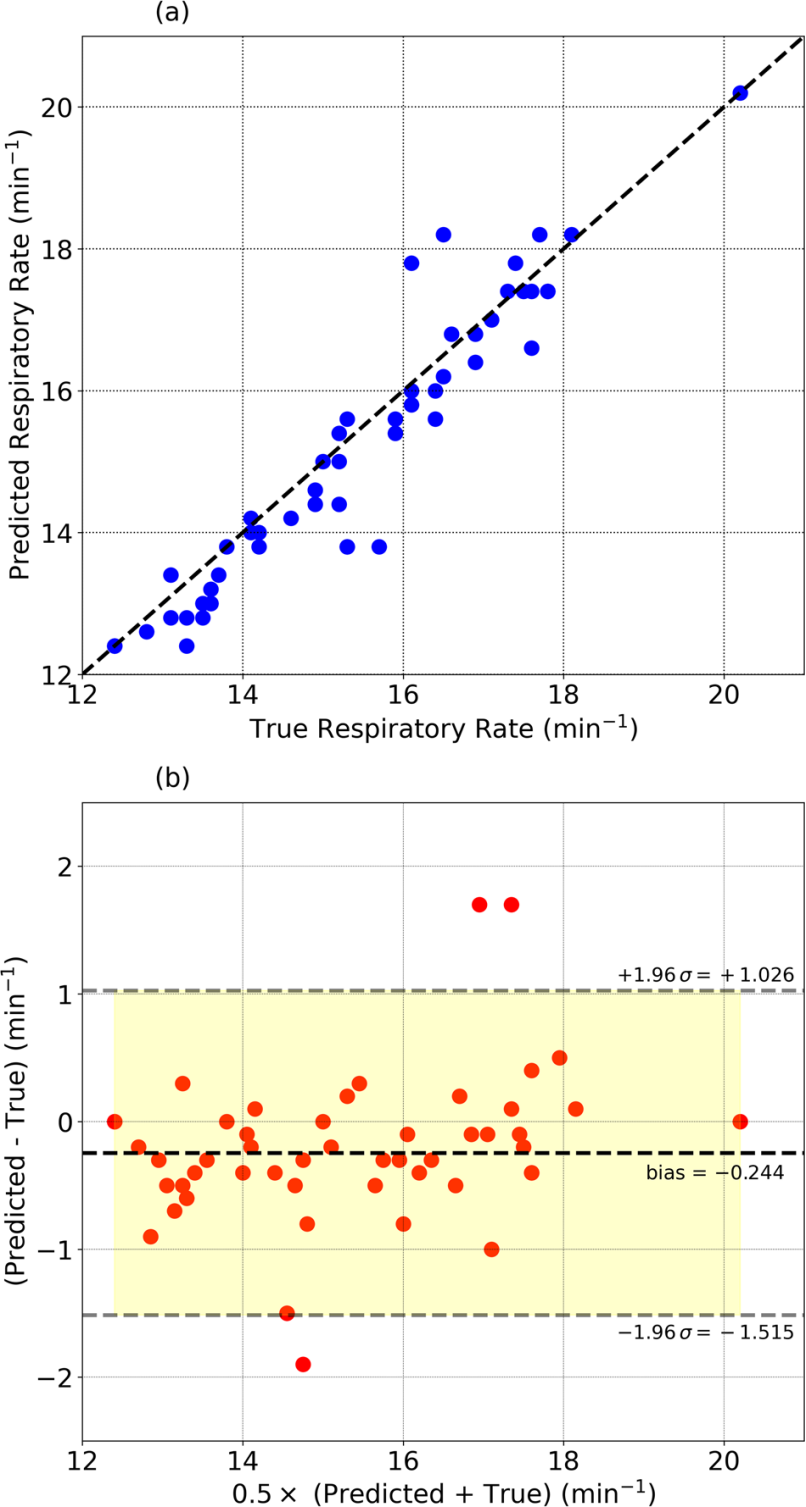
Comparison with ground truth measurements. **a** Respiratory rate estimated from the heart beat interval time series data compared to ground truth measurements. **b** shows the Bland-Altman plot comparing the true and predicted values. The bias (mean of predicted value–true value) is −0.24 min^−1^ (−1.67%). The RMS error = 0.65 min^−1^ and the mean absolute error = 0.46 min^−1^ (3.0%). The 95% region is shown in yellow.

Figure 8 shows the comparison between the true respiratory rate (nocturnal average) and the rate estimated from the peak of the heart beat interval power spectral density (nocturnal average). Plot (a) shows 52 measurements in the range (10 min^−1^, 26 min^−1^) with SNR ≥ 2.5, obtained from 28 individuals with apnea-hypopnea index < 30. The Pearson correlation coefficient *r* = 0.9515. Plot (b) shows the Bland-Altman plot of the difference in measurements (predicted value–true value) plotted against the average of the two. The bias (mean of the difference between predicted and true values) is −0.244 min^−1^(−1.67%), and the root mean squared error is 0.648 min^−1^(4.2%). The mean absolute error is found to be 0.460 min^−1^, and the mean absolute percentage error = 3.0%.

### Reporting summary

Further information on research design is available in the Nature Research Reporting Summary linked to this article.

## Supplementary information

Supplementary Information

Reporting Summary

## Data Availability

Fitbit’s privacy policy does not permit us to make the raw data or aggregate data available to third parties including researchers, outside of our web API Oauth 2.0 consent process. For specific questions, contact Fitbit at https://healthsolutions.fitbit.com/contact/.

## Code availability

We are unable to make our code publicly available. The code makes use of standard Python and Scala libraries.
