# Supplementary material for: Measurement of respiratory rate using wearable devices and applications to COVID-19 detection
Source: NPJ Digit Med. 2021 Sep 15;4:136. doi: 10.1038/s41746-021-00493-6 (PMC8443549; doi:10.1038/s41746-021-00493-6)
Supplement: Supplementary file 1 — Supplementary Information [file 41746_2021_493_MOESM1_ESM.pdf]

## Supplementary Text

Supplementary Table I shows the details of the 2 experiments conducted to validate the respiratory rate computations. Experiment A was conducted at Sleep Med in Columbia, SC, from Oct 17, 2019 to Nov 6, 2019. Experiment B was conducted remotely, by shipping equipment to the homes of participants, from March 9, 2020 to May 29, 2020. Participants in Experiment A wore Fitbit devices on both wrists, while participants in Experiment B wore a Fitbit device on one wrist only. We excluded participants with severe sleep apnea (Apnea-Hypopnea Index $\geq 30$). The columns are: (i) participant ID, (ii) age, (iii) sex, (iv) experiment (A or B), (v) night of measurement (1,2, or 3), (vi) apnea-hypopnea index, (vii) wrist (left, right, or unknown), (viii) predicted respiratory rate, and (ix) true respiratory rate.

### Validation of respiratory rate

| ID | Age (yr) | Sex (M/F) | Expt (A/B) | Night (1/2/3) | AHI | Wrist (L/R/-) | Pred. Rate ($min^{-1}$) | True Rate ($min^{-1}$) | ID | Age (yr) | Sex (M/F) | Expt (A/B) | Night (1/2/3) | AHI | Wrist (L/R/-) | Pred. Rate ($min^{-1}$) | True Rate ($min^{-1}$) |
|---|---|---|---|---|---|---|---|---|---|---|---|---|---|---|---|---|---|
| 1 | 35-39 | F | A | 1 | 9.2 | L | 16 | 16.4 | 13 | 45-49 | F | A | 1 | 1.5 | R | 14.4 | 14.9 |
| 1 | 35-39 | F | A | 1 | 9.2 | R | 15.6 | 16.4 | 14 | 35-39 | M | A | 1 | 2.7 | L | 14.4 | 15.2 |
| 2 | 55-59 | F | A | 1 | 17.3 | L | 15.6 | 15.9 | 14 | 35-39 | M | A | 1 | 2.7 | R | 15 | 15.2 |
| 2 | 55-59 | F | A | 1 | 17.3 | R | 15.4 | 15.9 | 15 | 45-49 | M | B | 1 | 13 | - | 13.8 | 13.8 |
| 3 | 50-54 | F | A | 1 | 1.6 | L | 12.4 | 12.4 | 15 | 45-49 | M | B | 2 | 13.2 | - | 14.2 | 14.6 |
| 3 | 50-54 | F | A | 1 | 1.6 | R | 12.4 | 12.4 | 16 | 50-54 | M | B | 2 | 4.2 | - | 18.2 | 18.1 |
| 4 | 65-69 | F | A | 1 | 5.3 | L | 13 | 13.5 | 17 | 30-34 | F | B | 1 | 25 | - | 17.8 | 16.1 |
| 4 | 65-69 | F | A | 1 | 5.3 | R | 12.8 | 13.5 | 17 | 30-34 | F | B | 2 | 12.1 | - | 16.8 | 16.6 |
| 5 | 55-59 | F | A | 1 | 10 | L | 13.8 | 14.2 | 17 | 30-34 | F | B | 3 | 14.6 | - | 17 | 17.1 |
| 5 | 55-59 | F | A | 1 | 10 | R | 14 | 14.2 | 18 | 35-39 | M | B | 1 | 4.8 | - | 13.2 | 13.6 |
| 6 | 55-59 | M | A | 1 | 26 | L | 13.4 | 13.1 | 18 | 35-39 | M | B | 2 | 5.6 | - | 13.4 | 13.7 |
| 6 | 55-59 | M | A | 1 | 26 | R | 12.8 | 13.1 | 19 | 50-54 | M | B | 1 | 7.6 | - | 17.4 | 17.5 |
| 7 | 55-59 | F | A | 1 | 7.5 | L | 20.2 | 20.2 | 19 | 50-54 | M | B | 2 | 9.8 | - | 17.4 | 17.3 |
| 7 | 55-59 | F | A | 2 | 26.3 | L | 17.4 | 17.6 | 20 | 70-74 | F | B | 1 | 18.5 | - | 15.4 | 15.2 |
| 7 | 55-59 | F | A | 2 | 26.3 | R | 16.6 | 17.6 | 21 | 45-49 | M | B | 1 | 20.1 | - | 16.4 | 16.9 |
| 8 | 50-54 | M | A | 1 | 0.6 | L | 13 | 13.6 | 21 | 45-49 | M | B | 2 | 17.2 | - | 16.8 | 16.9 |
| 8 | 50-54 | M | A | 1 | 0.6 | R | 13 | 13.6 | 22 | 50-54 | M | B | 2 | 14.4 | - | 16.2 | 16.5 |
| 9 | 50-54 | F | A | 1 | 16.9 | L | 17.4 | 17.8 | 23 | 50-54 | F | B | 1 | 4.6 | - | 12.4 | 13.3 |
| 9 | 50-54 | F | A | 1 | 16.9 | R | 17.4 | 17.8 | 23 | 50-54 | F | B | 2 | 5.8 | - | 12.8 | 13.3 |
| 10 | 45-49 | M | A | 1 | 27.1 | L | 14.2 | 14.1 | 24 | 35-39 | F | B | 1 | 14.2 | - | 17.8 | 17.4 |
| 10 | 45-49 | M | A | 1 | 27.1 | R | 14 | 14.1 | 24 | 35-39 | F | B | 2 | 12.4 | - | 18.2 | 17.7 |
| 11 | 40-44 | F | A | 1 | 2.6 | L | 15.8 | 16.1 | 25 | 40-44 | M | B | 1 | 13.8 | - | 18.2 | 16.5 |
| 11 | 40-44 | F | A | 1 | 2.6 | R | 16 | 16.1 | 26 | 50-54 | F | B | 1 | 12.5 | - | 15 | 15 |
| 12 | 40-44 | F | A | 1 | 5 | L | 13.8 | 15.3 | 26 | 50-54 | F | B | 2 | 12.6 | - | 15.6 | 15.3 |
| 12 | 40-44 | F | A | 1 | 5 | R | 13.8 | 15.3 | 27 | 45-49 | M | B | 2 | 19.3 | - | 12.6 | 12.8 |
| 13 | 45-49 | F | A | 1 | 1.5 | L | 14.6 | 14.9 | 28 | 65-69 | M | B | 1 | 6.3 | - | 13.8 | 15.7 |

Supplementary Table I: Details of experiments A and B. The columns are: participant ID, age, sex, experiment number (A or B), night of observation (1,2, or 3), apnea hypopnea index, wrist (left, right, or unknown), predicted respiratory rate, and true respiratory rate.
